# Comparison of the Effects of Normocapnia and Mild Hypercapnia on the Optic Nerve Sheath Diameter and Regional Cerebral Oxygen Saturation in Patients Undergoing Gynecological Laparoscopy with Total Intravenous Anesthesia

**DOI:** 10.3390/jcm10204707

**Published:** 2021-10-14

**Authors:** Chun-Gon Park, Wol-Seon Jung, Hee-Yeon Park, Hye-Won Kim, Hyun-Jeong Kwak, Youn-Yi Jo

**Affiliations:** Department of Anesthesiology and Pain Medicine, Gil Medical Center, Gachon University, Namdong-daero 774 beon-gil, Namdong-gu, Incheon 21565, Korea; chungony@gilhospital.com (C.-G.P.); cherish@gilhospital.com (W.-S.J.); hypark@gilhospital.com (H.-Y.P.); 18088@gilhospital.com (H.-W.K.)

**Keywords:** hypercapnia, lung-protective ventilation, Trendelenburg position, optic nerve sheath diameter, pneumoperitoneum, total intravenous anesthesia

## Abstract

Cerebral hemodynamics may be altered by hypercapnia during a lung-protective ventilation (LPV), CO_2_ pneumoperitoneum, and Trendelenburg position during general anesthesia. The purpose of this study was to compare the effects of normocapnia and mild hypercapnia on the optic nerve sheath diameter (ONSD), regional cerebral oxygen saturation (rSO_2_), and intraoperative respiratory mechanics in patients undergoing gynecological laparoscopy under total intravenous anesthesia (TIVA). Sixty patients (aged between 19 and 65 years) scheduled for laparoscopic gynecological surgery in the Trendelenburg position. Patients under propofol/remifentanil total intravenous anesthesia were randomly assigned to either the normocapnia group (target PaCO_2_ = 35 mmHg, *n* = 30) or the hypercapnia group (target PaCO_2_ = 50 mmHg, *n* = 30). The ONSD, rSO_2_, and respiratory and hemodynamic parameters were measured at 5 min after anesthetic induction (Tind) in the supine position, and at 10 min and 40 min after pneumoperitoneum (Tpp10 and Tpp40, respectively) in the Trendelenburg position. There was no significant intergroup difference in change over time in the ONSD (*p* = 0.318). The ONSD increased significantly at Tpp40 when compared to Tind in both normocapnia and hypercapnia groups (*p* = 0.02 and 0.002, respectively). There was a significant intergroup difference in changes over time in the rSO2 (*p* < 0.001). The rSO_2_ decreased significantly in the normocapnia group (*p* = 0.01), whereas it increased significantly in the hypercapnia group at Tpp40 compared with Tind (*p* = 0.002). Alveolar dead space was significantly higher in the normocapnia group than in the hypercapnia group at Tpp40 (*p* = 0.001). In conclusion, mild hypercapnia during the LPV might not aggravate the increase in the ONSD during CO_2_ pneumoperitoneum in the Trendelenburg position and could improve rSO_2_ compared to normocapnia in patients undergoing gynecological laparoscopy with TIVA.

## 1. Introduction

Lung protective ventilation (LPV) strategies to minimize ventilator-induced lung injuries are based on low tidal volume ventilation and permissive hypercapnia, which is a concomitant result of lung protective ventilation and an inherent element. LPV and permissive hypercapnia has been reported to reduce postoperative respiratory complications compared with non-protective ventilation in patients undergoing laparoscopic surgery [1,2]. Theoretically, increased blood carbon dioxide induces cerebral vasodilation, which might lead to increased cerebral blood volume (CBV) and intracranial pressure (ICP) with reduced cerebral perfusion pressure [3]. The combination of CO_2_ pneumoperitoneum and the steep Trendelenburg position for laparoscopic gynecological surgery can increase ICP [4,5] and alter CBV and cerebral blood flow (CBF) [6]. Changes in cerebral hemodynamics may affect not only cerebral perfusion pressure [7] but also cerebral oxygenation. In a prospective clinical study, the combination of pneumoperitoneum and the Trendelenburg position during laparoscopy significantly increased the ICP estimated by the optic nerve sheath diameter (ONSD), which was measured by ultrasonography and the transcranial Doppler-derived pulsatility index [8]. Another clinical study has reported a 12.5% increase in the ONSD during pneumoperitoneum in the steep Trendelenburg position for robot-assisted laparoscopic radical prostatectomy [9]. Lee et al. [10] reported that the regional cerebral oxygen saturation (rSO_2_) decreased after a change in the Trendelenburg position during gynecological laparoscopy.

Hypercapnia can abolish cerebral autoregulation during general anesthesia, and the extent may vary depending on the type of anesthetic agent used. In a previous clinical study, there was a significant difference in the threshold of the PaCO_2_ for impairing cerebral autoregulation between propofol and sevoflurane anesthesia (61 ± 4 mmHg vs. 56 ± 4 mmHg) [11]. Additionally, propofol-based total intravenous anesthesia (TIVA) could provide significantly lower ICP with improved hemodynamics than isoflurane-based inhalation anesthesia during emergency craniotomy [12].

To date, there have been no studies comparing the change in the ONSD between normocapnia and hypercapnia during pneumoperitoneum in the Trendelenburg position under TIVA. We hypothesized that hypercapnia, often accompanied by LPV, might increase the ONSD over normocapnia in the Trendelenburg position under propofol-based TIVA. The aim of this study was to compare the effects of normocapnia and mild hypercapnia on the ONSD, rSO_2_, and intraoperative respiratory parameters in patients undergoing laparoscopic gynecological surgery during TIVA.

## 2. Materials and Methods

This study was approved by the Institutional Review Board of the Gachon University Gil Hospital (GBIRB2020-248) and registered at cris.nih.go.kr, the clinical research information service run by the Korean government (KCT0005263). Written informed consent was obtained from all the participants prior to their registration. Sixty patients scheduled for elective total laparoscopic hysterectomy were included. Patients aged between 19 and 65 years with an American Society of Anesthesiologists physical status of 1 or 2 were included. Patients with a history of congestive heart failure, coronary artery obstructive disease, uncontrolled hypertension, uncontrolled diabetes mellitus, cerebrovascular disease, previous hemorrhage or trauma in the brain, active pulmonary infection, obstructive or interstitial pulmonary disease, glaucoma, previous history of ophthalmic surgery, and body mass index greater than 25 kg/m^2^ were excluded. Enrolled patients were randomly assigned to either the normocapnia group (*n* = 30) or the hypercapnia group (*n* = 30), using the randomization function in Excel 2013 (Microsoft Office, Redmond, WA, USA).

No sedatives or analgesics were provided prior to surgery for premedication. A non-invasive blood pressure monitor, pulse oximeter, electrocardiograph, and bispectral index (BIS) were employed prior to anesthetic induction. The rSO_2_ was measured continuously using the INVOS^TM^ Cerebral/Somatic Oximeter 5100 (Covidien, Dublin, Ireland) with the cerebral oximeter sensors on both frontotemporal areas. Propofol (Schnider’s pharmacokinetic model; target blood concentration 2.5–4.0 ug/mL) and remifentanil (Minto’s pharmacokinetic model; target blood concentration 2.5–4.0 ng/mL), set by a target-controlled infusion device (Orchestra; Fresenius Kabi, Bad Homburg, Germany) were administered for anesthetic induction and maintenance for target BIS of 40–60. Rocuronium 0.8 mg/kg was injected to facilitate tracheal intubation. After anesthetic induction, the mechanical ventilator was set in volume-controlled mode with a tidal volume of 6 mL/kg of ideal body weight (0.919 × (height in cm − 152.4) + 45.5), an inspiratory to expiratory (I/E) ratio of 1:1, a positive end-expiratory pressure (PEEP) of 5 cmH_2_O, Tpause of 10 s, and an inspired oxygen fraction (FiO_2_) of 0.5 with oxygen and medical air. The respiratory rate was adjusted to a target end-tidal carbon dioxide tension (E_T_CO_2_) of 35 ± 2 mm Hg for 5 min after anesthetic induction. Subsequently, the respiratory rate was adjusted to an E_T_CO_2_ of 28 ± 2 mmHg (target PaCO_2_ 35 mmHg) for the normocapnia group and 43 ± 2 mmHg (target PaCO_2_ 50 mmHg) for the hypercapnia group. For the laparoscopic procedure, abdominal insufflation with CO_2_ gas at 15 mmHg and a 30° Trendelenburg position were adopted. We obtained ultrasound images of the optic nerve sheath diameter (ONSD), using 6–15 Hz high-frequency ultrasound probe (Z.one^®^ ultrasound system, Zonare medical system, Mountain View, CA, USA). The probe with lubricant was placed gently above the disposable eye protection patch, which covered the patient’s closed eyes. Axial images of the orbit were acquired in the plane of the optic nerve, and the ONSD was the distance between the outer border of the hyperechogenic line at 3 mm behind the optic disc. The measurement was performed three times in both eyes at a single time point, and the mean values were calculated. The measurements were performed 3 mm below the margin of the eyeball and the hypoechoic bulb. An investigator (Y.-Y.J.), who was blinded to group allocation, measured the ONSD at 5 min after anesthesia induction in the supine position (Tind) and at 10 min and 40 min after pneumoperitoneum (Tpp10 and Tpp40, respectively) in the Trendelenburg position. Arterial blood gas analysis was performed by single radial arterial puncture at two time points (Tind and Tpp40). Mean arterial pressure, heart rate, and recovery scores were obtained in the postanesthetic care unit (PACU).

Hemodynamic variables (mean arterial pressure and heart rate) and respiratory parameters (E_T_CO_2_, peak and plateau airway pressure (Ppeak and Pplat)), tidal volume (TV), respiratory rate (RR), PEEP, BIS, and rSO_2_ were recorded. Dynamic and static lung compliance (Cdyn and Cstat) were calculated using the equation Cdyn = TV/(Ppeak − PEEP); Cstat = TV/(Pplat − PEEP). The mechanical power (MP) was calculated using the equation MP = 0.098 × RR × TV × [Ppeak − 0.5(Pplat − PEEP)] [13]. The alveolar dead space fraction (Vd/Vt) was calculated using the equation Vd/Vt = 1.135 × (PaCO_2_ − E_T_CO_2_)/(PaCO_2_ − 0.005) × 100 [14].

The primary outcome was the difference in the ONSD during pneumoperitoneum, and the secondary outcomes were rSO_2_ and intraoperative respiratory parameters. The sample size was calculated based on a previous study [15], which measured the ONSD according to changes in E_T_CO_2_ at normocapnia and hypercapnia. Twenty-four patients were required per group for a power of 90% and an error of 0.05, and assuming possible dropout, a total of 60 patients were recruited [16].

SPSS version 19.0 (SPSS Inc., Chicago, IL, USA) was used for statistical analysis. Data were presented as mean ± SD or as the number of patients (%) as median [interquatile range]. The Kolmogorov–Smirnov test was performed to assess the normality of the continuous variable distribution. An independent *t*-test or the Mann–Whitney U test for continuous variables and the x^2^-test or Fisher’s exact test for categorical variables were used as appropriate. Changes in variables over time were analyzed using a repeated-measures analysis of variance (ANOVA). Statistical significance was set at *p* < 0.05.

## 3. Results

**Figure 1:**
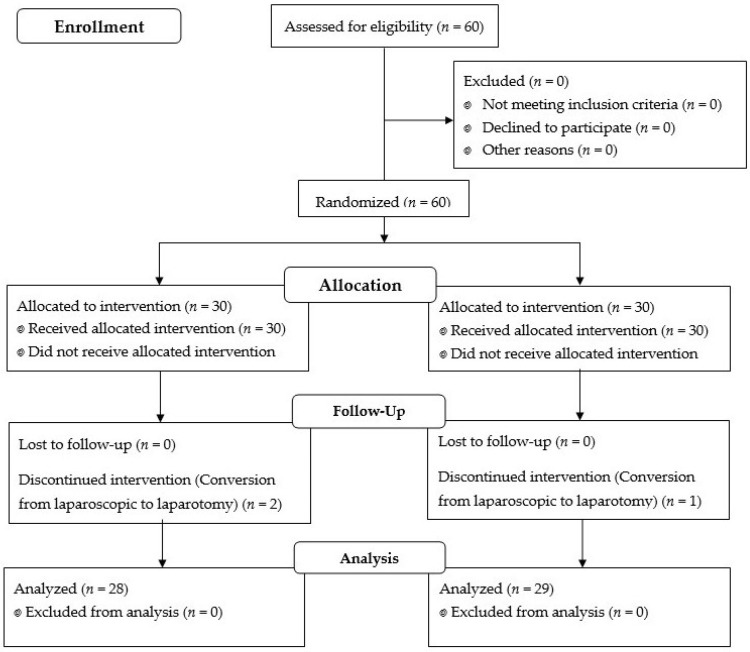
Flow diagram of patient allocation.

Among the 60 enrolled patients, two in the normocapnia group and one in the hypercapnia group discontinued the allocated intervention due to a conversion of the surgery from laparoscopy to laparotomy during the surgery (Figure 1).

Demographic data were similar between the groups. Intraoperative data, including surgical time and consumption of propofol and remifentanil, were similar between the groups (Table 1).

**Figure 2:**
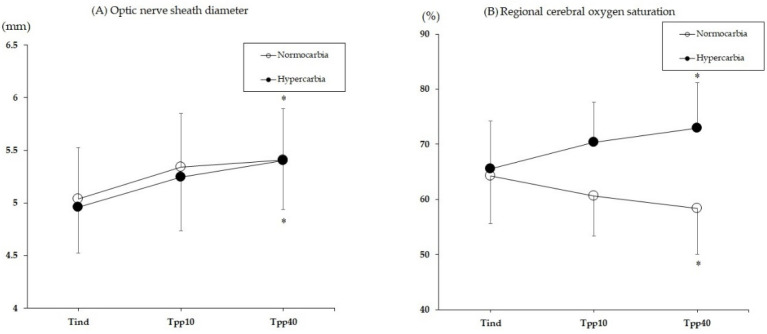
The changes in the optic nerve sheath diameter (**A**) and the regional cerebral oxygen saturation (**B**) during the surgery. Tind, 10 min after the anesthesia induction; Tpp10 and 40, 10 and 40 min after the induction of pneumoperitoneum, respectively. * *p* < 0.05 vs. Tind within the group.

The changes in the rSO_2_ and the ONSD are illustrated in Figure 2. There was no significant intergroup difference in change over time in the ONSD group (*p* = 0.318), but the ONSD increased significantly over time in both groups (*p* < 0.001). The ONSD was increased by 7.7 ± 8.1% in the normocapnia group (*p* = 0.02), and by 5.9 ± 6.3% in the hypercapnia group (*p* = 0.002) at Tpp40 compared to Tind. At Tpp40, the number of patients with an ONSD > 5.5 mm was 10 in the normocapnia group and 7 in the hypercapnia group (*p* = 0.395). There was a significant intergroup difference in changes over time in the rSO_2_ group (*p* < 0.001). The rSO_2_ was decreased by 8.2 ± 12.6% in the normocapnia group (*p* = 0.01), whereas it increased by 11.7 ± 8.3% in the hypercapnia group (*p* = 0.002) at Tpp40 compared with Tind. There were three patients in the normocapnia group with a ≥20% reduction in the rSO_2_ during anesthesia.

The intraoperative respiratory parameters are shown in Table 2. There were no significant intergroup differences in the changes over time in Ppeak, Pplat, Cdyn, and Cstat (*p* = 0.948, 0.455, 0.654, and 0.903, respectively). The airway pressure (Ppeak and Pplat) and respiratory compliance (Cdyn and Cstat) changed significantly at Tpp10 and Tpp40 compared with Tind in both groups (all *p* values < 0.001). There were significant intergroup differences in the changes over time in RR and MP (all *p* values < 0.001). RR and MP increased significantly at Tpp10 and Tpp40 compared with Tind in the normocapnia group (all *p* values < 0.001). RR decreased significantly at Tpp10 compared with Tind in the hypercapnia group (*p* = 0.025).

E_T_CO_2_ and arterial blood gas analysis data are presented in Table 3. The E_T_CO_2_ decreased significantly in the normocapnia group (*p* = 0.001) but increased significantly in the hypercapnia group (*p* < 0.001) at Tpp40 compared with Tind. The pH was significantly lower in the hypercapnia group than in the normocapnia group at Tpp40 (*p* < 0.001). At Tpp40, the pH decreased significantly compared with Tind in the hypercapnia group (*p* < 0.001). Vd/Vt was significantly higher in the normocapnia group than in the hypercapnia group at Tpp40 (*p* = 0.001).

There were no significant intergroup differences in the changes in the mean arterial pressure and heart rate (*p* = 0.057 and 0.113, respectively) during the surgery (data not shown).

In the postanesthetic care unit, the mean arterial pressure (normocapnia vs. hypercapnia; 95.9 ± 14.7 mmHg vs. 91.9 ± 13.8 mmHg, *p* = 0.302) and heart rate (72.6 ± 12.5 beats/min vs. 74.6 ± 12.0 beats/min, *p* = 0.545) were similar between the groups. Modified Aldrete score were similar between the groups (10 [9-10] vs. 10 [9-10], *p* = 0.842). No patient experienced delirium or desaturation under SaO_2_ < 90%.

## 4. Discussion

This study demonstrated that mild hypercapnia for LPV might not aggravate the increase in the ONSD during CO_2_ pneumoperitoneum in the Trendelenburg position and could improve the rSO_2_ and respiratory parameters compared to normocapnia in patients undergoing gynecological laparoscopy with TIVA.

In this study, the combination of CO_2_ pneumoperitoneum and the Trendelenburg position significantly increased the ONSD, regardless of the study group. This is consistent with the results of a previous study, which reported a significant increase in the ONSD during CO_2_ pneumoperitoneum with a steep Trendelenburg position for laparoscopic prostatectomy [9]. It is a widely known fact that hypercapnia induces cerebral vasodilation and increases ICP, and according to the previous study, the ONSD of 6.5 kPa hypercapnia increased from 4.2 ± 0.7 mm to 4.8 ± 0.8 mm compared to normocapnia [15]. However, this study showed a lack of differences in the ONSD between the hypercapnia and normocapnia groups. This result may be associated with the propofol-based TIVA used for general anesthesia in this study.

In an earlier study that compared the effect of anesthetic agents on the ONSD for laparoscopic prostatectomy, propofol was shown to attenuate increase in the ONSD compared to sevoflurane [17]. Although the ONSD was gradually increased during pneumoperitoneum with a steep Trendelenburg position in both propofol and sevoflurane anesthesia, the ONSD in the propofol group was smaller than that in the sevoflurane group at all study time points measured during pneumoperitoneum, and there was also a significant difference at 60 min after pneumoperitoneum [17]. Even mild hypercapnia can impair cerebral autoregulation during a general anesthesia. Meanwhile, in a study by McCulloch et al., it was found that propofol can attenuate the effect of hypercapnia on cerebral autoregulation compared to sevoflurane anesthesia if the mean arterial pressure is constant [11]. In addition, the threshold of PaCO_2_ to impair cerebral autoregulation has been reported to be 50–66 mmHg, and the average threshold was significantly higher in the propofol group than in the sevoflurane group (61 ± 4 mmHg vs. 56 ± 4 mmHg) [11]. Thus, cerebral autoregulation was well preserved even in the hypercapnia group in this study because the target PaCO_2_ in the hypercapnia group was 50 mmHg, which was lower than the threshold level of the propofol group.

The decrease in the rSO_2_ in the normocapnia group during pneumoperitoneum was consistent with a previous study on gynecological laparoscopy by Lee et al. [10]. They suggested that the rSO_2_ was decreased by the ICP increase and the CPP decrease due to the Trendelenburg position rather than pneumoperitoneum [10]. This study showed a trend of decreasing rSO_2_ in the normocapnia group, whereas a trend of increasing rSO_2_ in the hypercapnia group. There was no difference between the two groups in the ONSD used to predict the ICP in this study, so if we explain these results as the relationship between the CBF and the PaCO_2_, the explanation of the results in the ONSD and the interpretation of the results in the rSO_2_ may end up being conflicting. Wong et al. demonstrated a stable increase in the rSO_2_ in the targeted mild hypercapnia (PaCO_2_ 45–55 mmHg) group and a decrease in the rSO_2_ in the targeted normocapnia (PaCO_2_ 35–40 mmHg) group in patients undergoing major surgery during similar mean arterial pressure, PaO_2_, and Hb levels [18]. Murphy et al. also reported higher rSO_2_ during the beach chair position in the mild hypercapnia group than in the normocapnia group [19]. Since PaCO_2_ is a strong regulator of CBF independent of cerebral autoregulation [20], increased CBF could contribute to the increased rSO_2_ in the hypercapnia group in this study.

Hypercapnia is a potent factor in the development of pulmonary vasoconstriction [21]. In the previous evaluation of vascular response to hypercapnia in healthy lungs, the response to the PaO_2_ was more dominant than the PaCO_2_ at lower regional ventilation/perfusion (V/Q) ratios (<0.65), and the response to PaCO_2_ was more dominant than PaO_2_ at higher V/Q ratios (>0.65) [22]. In this study, the alveolar dead space fraction was significantly increased during pneumoperitoneum in the Trendelenburg position compared to the baseline value in the normocapnia group but not in the hypercapnia group. The changes in the normocapnia group were consistent with the results of a previous study [23]. During the lung-protective ventilation, the dead space to tidal volume ratio was significantly increased after pneumoperitoneum combined with the Trendelenburg position in an earlier study besides various PEEP levels [23]. However, enhanced hypercapnia-induced pulmonary vasoconstriction might attenuate the increase in dead space. Similar results were found in the sitting position under general anesthesia. After changing position from the supine to sitting position, the dead space ventilation ratio increased significantly from 6.3 ± 11.3% to 12.7 ± 9.4% in the normocapnia group and changed from 5.2 ± 8.9% to 7.3 ± 8.9% in the hypercapnia group, showing a larger increase in the hypercapnia group [24]. These study results suggest that permissible hypercapnia, rather than the change in position, can attenuate the increase in dead space that occurs after general anesthesia.

MP is energy delivered from the mechanical ventilator to the respiratory system, and an increase in MP is known to be a predictor of ventilator-induced lung injury [25]. In our study, MP was significantly lower in the hypercapnia group than in the normocapnia group during pneumoperitoneum in the Trendelenburg position. Considering the formula for calculating MP, it can be considered that increasing the respiratory rate to maintain normocapnia, as in our study, resulted in an increase in MP in the normocapnia group during CO_2_ pneumoperitoneum. Although there is insufficient clinical evidence to simply compare the meaning of ventilator-induced lung injury or mechanical power in patients under general anesthesia with healthy lungs and critically ill patients with lung injury receiving ventilator care, it is necessary to consider the fact that the respiratory rate, which is often neglected, may have a negative effect on the lungs or induce ventilator-induced lung injury [26]. Therefore, it is worth noting that the load on the lungs can be reduced by allowing mild hypercapnia rather than excessively setting the respiratory rate to maintain normocapnia.

This study had several limitations. First, we only included patients aged between 19 and 65 years with an ASA physical status of 1 or 2. In the elderly, vascular responses to hypercapnia or hypoxia are different from those in young individuals, and underlying cerebrovascular or cardiopulmonary morbidity may also contribute to changes in outcome [27]. Therefore, our findings cannot be generalized and applied to all age groups. Second, we estimated the change in the ICP by measuring the ONSD. Although measuring the ONSD has been proven as a valid alternative to invasive ICP measurement, the intraventricular catheter measurement is still known as the gold standard for ICP monitoring [28]. At last, the preoperative value of the rSO_2_ could be very informative since the previous study has reported more than 20% decrease in rSO_2_ compared to the preoperative value, which is a very important cause of postoperative delirium [29]. Unfortunately, preoperative and intraoperative values cannot be compared in this study because we did not record the rSO_2_ value prior to anesthetic induction. In this study, there were three patients in the normocapnia group with a ≥20% reduction in rSO_2_ during anesthesia and none of patients experienced delirium in the postanesthetic care unit.

In conclusion, mild hypercapnia during LPV might not aggravate the increase in the ONSD during CO_2_ pneumoperitoneum in the Trendelenburg position and could improve the rSO_2_ compared to normocapnia in patients undergoing gynecological laparoscopy with TIVA.

## Figures and Tables

**Table 1:** Patient characteristics and perioperative data.

	Normocapnia (*n* = 28)	Hypercapnia (*n* = 29)	*p* Value
Age (years)	48 ± 5	48 ± 7	0.959
Body mass index (kg/m^2^)	24 ± 3	25 ± 3	0.168
Anesthesia time (min)	94 ± 31	103 ± 42	0.360
Operation time (min)	65 ± 30	74 ± 42	0.359
Pneumoperitoneum time (min)	53 ± 24	60 ± 35	0.343
Total infused propofol (mg)	676 ± 227	754 ± 249	0.474
Total infused remifentanil (ug)	587 ± 220	633 ± 249	0.474

Values are presented as mean ± SD or the number of patients.

**Table 2:** Changes of respiratory parameters during surgery.

Variables	Group	Tind	Tpp10	Tpp40
Ppeak (cmH_2_O)	Normocapnia	14 ± 4	21 ± 4 *	21 ± 4 *
	Hypercapnia	13 ± 2	20 ± 3 *	19 ± 3 *
Pplat (cmH_2_O)	Normocapnia	11 ± 4	17 ± 4 *	17 ± 4 *
	Hypercapnia	11 ± 2	17 ± 3 *	17 ± 4 *
RR (breaths/min)	Normocapnia	15 ± 3	20 ± 4 *	20 ± 4 *
	Hypercapnia	14 ± 4	11 ± 2 *	12 ± 2
Cdyn (l/cmH_2_O)	Normocapnia	36 ± 10	21 ± 5 *	21 ± 5 *
	Hypercapnia	37 ± 9	22 ± 4 *	23 ± 7 *
Cstat (l/cmH_2_O)	Normocapnia	61 ± 41	28 ± 9 *	27 ± 8 *
	Hypercapnia	58 ± 25	28 ± 9 *	28 ± 12 *
MP (J/min)	Normocapnia	5 ± 1	9 ± 2 *	9 ± 2 *
	Hypercapnia	4 ± 2	5 ± 1	5 ± 1

Values are presented as mean ± SD. Tind, 10 min after anesthesia induction; Tpp 10 and 40, 10, and 40 min after the induction of pneumoperitoneum; Ppeak, peak airway pressure; Pplat, plateau airway pressure; RR, respiratory rate; Cdyn, dynamic effective compliance; Cstat static effective compliance; MP, mechanical power. * *p* < 0.05 vs. Tind.

**Table 3:** Changes of E_T_CO_2_ and arterial blood gas analysis.

Variables	Group	Tind	Tpp40
E_T_CO_2_ (mmHg)	Normocapnia	34 ± 4	30 ± 4 *
	Hypercapnia	36 ± 5	44 ± 3 *
pH	Normocapnia	7.44 ± 0.03	7.42 ± 0.05
	Hypercapnia	7.42 ± 0.05	7.33 ± 0.05 *^,^^†^
PaO_2_ (mmHg)	Normocapnia	257 ± 61	202 ± 54 *
	Hypercapnia	263 ± 88	206 ± 48 *
PaCO_2_ (mmHg)	Normocapnia	37 ± 4	38 ± 5
	Hypercapnia	40 ± 6	50 ± 6 *^,^^†^
Vd/Vt (%)	Normocapnia	12 ± 9	23 ± 8 *
	Hypercapnia	13 ± 9	16 ± 6 *^,^^†^

Values are presented as mean ± SD. Tind, 10 min after anesthesia induction; Tpp40, 40 min after the induction of pneumoperitoneum; E_T_CO_2_, end-tidal carbon dioxide tension; Vd/Vt alveolar dead space ratio. * *p* < 0.05, vs. Tind within the group. ^†^ *p* < 0.05, vs. normocapnia group.

## Data Availability

Data is contained within the article.
